# Extraction of a perigastric foreign body using a lumen-apposing metal stent

**DOI:** 10.1055/a-2318-2761

**Published:** 2024-05-29

**Authors:** Félix Corre, Marine Carpentier-Pourquier, Juliette Leroux, Romain Coriat, Stanislas Chaussade, Arthur Belle, Maximilien Barret

**Affiliations:** 1 Department of Gastroenterology, Digestive Oncology, and Endoscopy, Cochin Hospital, Paris, France

Lumen-apposing metal stents (LAMS) were initially developed to drain perigastric pancreatic necrotic collections and perform endoscopic necrosectomy.

**Fig. 1:**
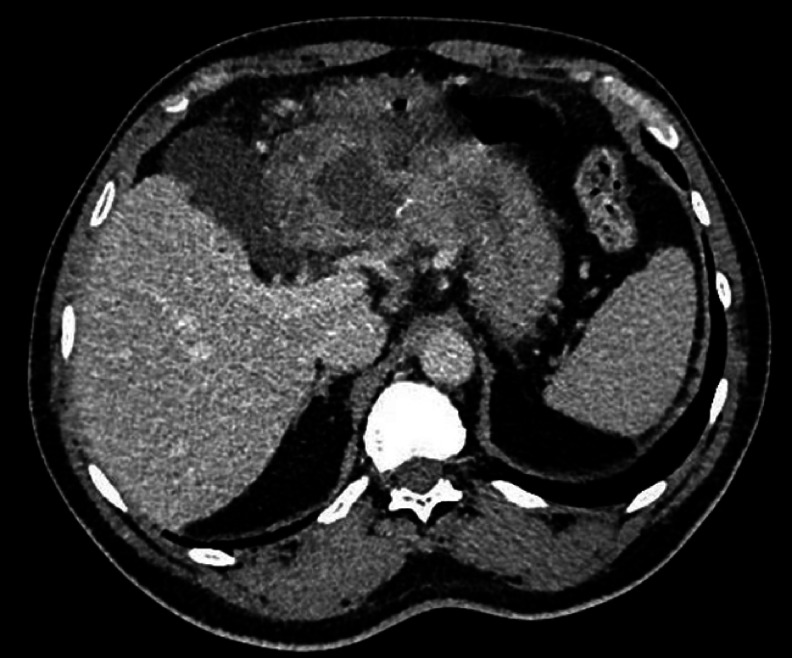
Initial CT scan showing an antral perigastric collection measuring 4 cm in diameter and containing a hyperdense linear image suggestive of a bone or fishbone foreign body.

**Fig. 2:**
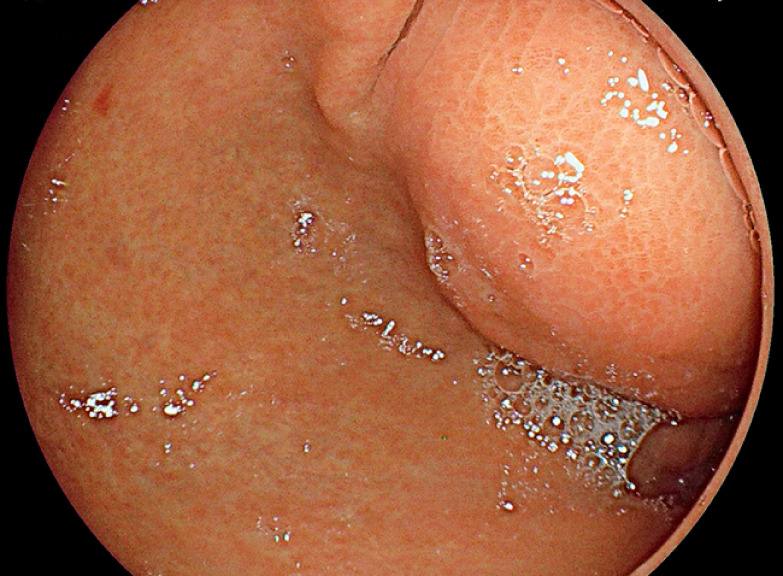
Upper gastrointestinal endoscopy showing a bulging collection in the prepyloric antrum.

**Fig. 3:**
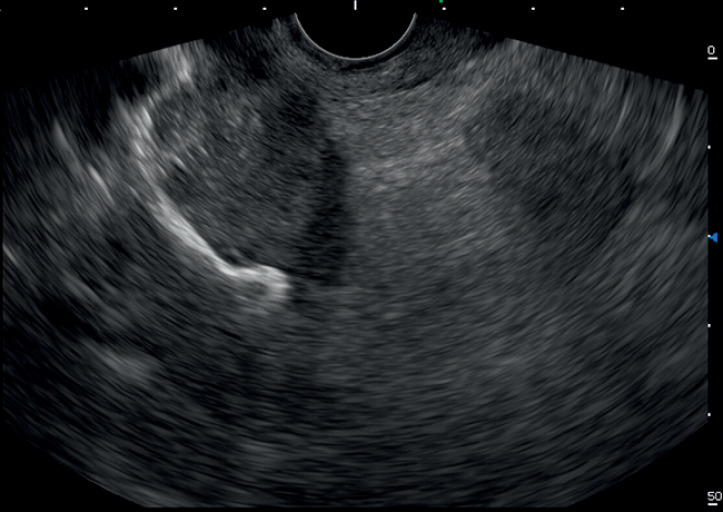
Cystogastrostomy using a 15 × 15 mm lumen-apposing metal stent (LAMS).

**Fig. 4:**
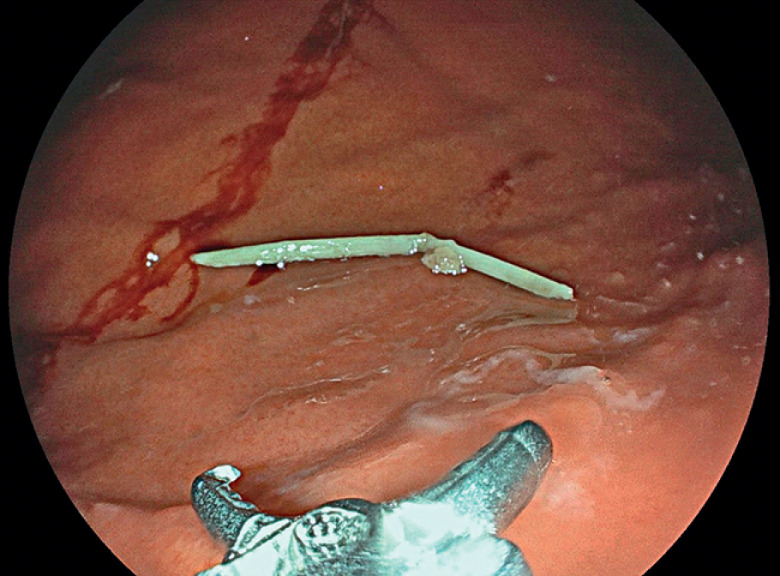
Fishbone extraction through the LAMS using foreign-body forceps.

We report the case of a man hospitalized for a 4-cm infected antral perigastric collection secondary to a fishbone that had perforated the gastric antrum ( Fig. 1 , Video 1 ). Upper gastrointestinal endoscopy showed a bulging collection in the prepyloric antrum ( Fig. 2 ). Given the location of the collection, its size, and the presence of a foreign body within it, we first performed endoscopic cystogastrostomy using a 15×15 mm LAMS ( Fig. 3 ). A second upper gastrointestinal endoscopy was performed 7 days later to extract the fishbone through the LAMS ( Fig. 4 ). The procedure was successful. No complication occurred.

Extraction of a perigastric foreign body using a lumen-apposing metal stent.Video 1

This endoscopic minimally invasive approach avoided surgery, and the patient was discharged from hospital the day after the fishbone extraction. The 6-month follow-up period was uneventful.

Endoscopy_UCTN_Code_TTT_1AO_2AL

